# *Coxiella burnetii* Genotyping

**DOI:** 10.3201/eid1108.041354

**Published:** 2005-08

**Authors:** Olga Glazunova, Véronique Roux, Olga Freylikman, Zuzana Sekeyova, Ghislain Fournous, Judith Tyczka, Nikolai Tokarevich, Elena Kovacova, Thomas J. Marrie, Didier Raoult

**Affiliations:** * Unité des Rickettsies, Marseille, France;; † Pasteur Institute of Epidemiology and Microbiology, Saint Petersburg, Russia;; ‡ Institute of Virology SAS, Bratislava, Slovak Republic;; § University of Giessen, Giessen, Germany;; ¶ University of Alberta Department of Medicine, Edmonton, Alberta, Canada

**Keywords:** Coxiella burnetii, Q fever, Phylogeny, Bacterial typing, DNA sequence analysis

## Abstract

*Coxiella burnetii* is a strict intracellular bacterium with potential as a bioterrorism agent. To characterize different isolates of *C*. *burnetii* at the molecular level, we performed multispacer sequence typing (MST). MST is based on intergenic region sequencing. These regions are potentially variable since they are subject to lower selection pressure than the adjacent genes. We screened 68 spacers in 14 isolates and selected the 10 that exhibited the most variation. These spacers were then tested in 159 additional isolates obtained from different geographic areas or different hosts or were implicated in different manifestations of human disease caused by *C*. *burnetii*. The sequence analysis yielded 30 different allelic combinations. Phylogenic analysis showed 3 major clusters. MST allows easy comparison and exchange of results obtained in different laboratories and could be a useful tool for identifying bacterial strains.

## Abstract 2

Multispacer sequence typing is the first reliable method for typing *Coxiella burnetii* isolates.

*Coxiella burnetii* is a strict intracellular microorganism, included in the γ subdivision of the Proteobacteria phylum (1). It is found in close association with arthropod and vertebrate hosts, and it causes Q fever in humans and animals. Cattle, goats, and sheep are the primary reservoirs of human infection. In humans, the disease may appear in 2 forms, acute and chronic (2). Acute Q fever may be asymptomatic or appear as atypical pneumonia, granulomatous hepatitis, or self-limited febrile illness. In some persons, the immune system is unable to control the infection and chronic Q fever occurs. The manifestations of chronic Q fever are endocarditis, hepatitis, osteomyelitis, or infected aortic aneurysms. *C*. *burnetii* is highly infectious by the aerosol route and can survive for long periods in the environment.

Previous studies have shown that *C*. *burnetii* isolates differed respect to their plasmid type (QpH1, QpRS, QpDG, and QpDV) (3–6), lipopolysaccharide profiles (7), and analysis of endonuclease-digested DNA separated by sodium dodecyl sulfate-polyacrylamide gel electrophoresis (8) or pulsed-field gel electrophoresis (PFGE) (9–11). Differentiation was also obtained by sequence determination of the isocitrate dehydrogenase gene (12), *com1* gene, and *mucZ* gene, which was renamed *djlA* when the whole genome of *C*. *burnetii* was sequenced (13,14).

Several other methods have been used to type different isolates of the same species, in particular, multilocus enzyme electrophoresis (15) and multilocus sequence typing (MLST) (16). Many bacterial species have been studied by using these approaches (17–19).

Recently, the whole genome of the *C*. *burnetii* Nine Mile strain was sequenced (14). We decided to investigate parts of the genome located between 2 open reading frames (ORFs) because they are considered potentially variable since they are subject to lower selection pressure than the adjacent genes. The 16S/23S ribosomal spacer region has been widely used to genotype bacteria (20–23). We investigated the utility of multispacer sequence typing (MST) with 173 *C*. *burnetii* isolates. After screening, we selected 10 variable spacers and showed that the combination of the different sequences allowed us to characterize 30 different genotypes. Phylogenetic analysis inferred from compiled sequences characterized 3 monophyletic groups, which could be subdivided into different clusters.

## Methods

### Bacterial Strains

The *C*. *burnetii* strains included in this study are listed in Table A1. All the strains were propagated on Vero cell monolayers (ATCC CRL 1587). Minimal essential medium (MEM) (Invitrogen, Cergy-Pontoise, France) supplemented with 4% fetal bovine serum (Invitrogen) and 1% L-glutamine (Invitrogen) was used for cultivation. Infected cells were maintained in a 5% CO_2_ atmosphere at 35°C. *C*. *burnetii* cells were harvested, pelleted, resuspended in 200 μL MEM, and mixed with 500 μL Chelex 100 20% (Bio-Rad, Ivry sur Seine, France). The preparation was boiled for 30 min, centrifuged at 10,000 × *g* for 30 min (24), and the supernatant containing DNA was transferred to a clean Eppendorf tube and stored at 4°C or –20°C.

### Multispacer Sequence Typing

The whole genome of *C*. *burnetii* was accessible in the NCBI server (GenBank NC 002971). We kept spacers that were 300–700 bp in length. Primers were chosen in neighboring genes to allow polymerase chain reaction (PCR) amplification at 57°C and are listed in Table 1. Each PCR was carried out in a T3 Thermocycler Biometra (Biolabo, Archamps, France). Two microliters of the DNA preparation was amplified in a 50-μL reaction mixture containing 200 μmol/L of each primer, 200 μmol/L (each) dATP, dCTP, dGTP, and dTTP (Invitrogen), 1.5 U Taq DNA polymerase (Roche, Meylan, France) in 1× Taq buffer. Amplifications were carried under the following conditions: initial denaturation of 10 min at 95°C, followed by 37 cycles of denaturation for 30 s at 95°C, annealing for 30 s at 57°C, and extension for 1 min at 72°C. PCR products were purified and sequenced as previously described (25).

**Table 1:** Primers used for PCR amplification and sequencing of Coxiella burnetii gene spacers

Spacer name	ORF	Nucleotide sequence (5´–3´)*	Amplified fragment length (bp)
Cox2	Hypothetical protein	Cox20766 CAACCCTGAATACCCAAGGA	397
	Hypothetical protein	Cox21004 GAAGCTTCTGATAGGCGGGA	
Cox5	Sulfatase domain protein	Cox77554 CAGGAGCAAGCTTGAATGCG	395
	Entericidin, putative	Cox77808 TGGTATGACAACCCGTCATG	
Cox18	Ribonuclease H	Cox283060 CGCAGACGAATTAGCCAATC	557
	DNA polymerase III, epsilon subunit	Cox283490 TTCGATGATCCGATGGCCTT	
Cox20	Hypothetical protein	Cox365301 GATATTTATCAGCGTCAAAGCAA	631
	Hypothetical protein	Cox365803 TCTATTATTGCAATGCAAGTGG	
Cox22	Hypothetical protein	Cox378718 GGGAATAAGAGAGTTAGCTCA	383
	Amino acid permease family protein	Cox378965 CGCAAATTTCGGCACAGACC	
Cox37	Hypothetical protein	Cox657471 GGCTTGTCTGGTGTAACTGT	463
	Hypothetical protein	Cox657794 ATTCCGGGACCTTCGTTAAC	
Cox51	Replicative DNA helicase, intein-containing	Cox824598 TAACGCCCGAGAGCTCAGAA	674
	Conserved hypothetical protein – Uridine kinase	Cox825124 GCGAGAACCGAATTGCTATC	
Cox56	OmpA-like transmembrane domain protein	Cox886418 CCAAGCTCTCTGTGCCCAAT	479
	Conserved hypothetical protein	Cox886784 ATGCGCCAGAAACGCATAGG	
Cox57	Rhodanese-like domain protein	Cox892828 TGGAAATGGAAGGCGGATTC	617
	Hypothetical protein	Cox893316 GGTTGGAAGGCGTAAGCCTTT	
Cox 61	Dioxygenase, putative	Cox956825 GAAGATAGAGCGGCAAGGAT	611
	Hypothetical protein	Cox957249 GGGATTTCAACTTCCGATAGA	

*The numbers are beginning or end locations of the genes where the primers were chosen.

PCR products were cloned in PGEM-T Easy Vector (Promega, Charbonnières, France) according to the manufacturer's instructions. Ten clones were cultivated in LB medium (USB, Cleveland, OH, USA) overnight, and PCR and sequencing were performed as described previously.

### Plasmid Sequence Type, *com1* Type, and *djlA* Type Determination

PCR for QpH1 and QpRS sequence plasmids were performed with the primers previously described QpH11/12 and QpRS01/02 (5). PCR was carried out as described for MST, except that annealing temperature was 55°C and cycle number was 35. PCR primers for QpDV and QpRS sequence plasmid amplification were chosen after comparison of the entire sequence of the 2 plasmids. The primers were QpDV1f and QpDV1r. PCR amplification was carried out at 63°C for 30 cycles. PCR was performed as previously described for *com1* and *djlA* (13) (Table A2).

## Data Analysis

Statistical analyses were performed by using the chi-square test in the program EpiInfo 6 (26). The spacer sequences were compiled and aligned by using the multisequence alignment program ClustalX (1.8). The phylogenetic relationships between the *C*. *burnetii* isolates were determined by using Mega version 2.0 (27). A matrix of pairwise differences in allele profiles was constructed, and the similarities between the allelic profiles of the isolates were assessed by cluster analysis using the unweighted pair-group method with arithmetic mean (UPGMA). Another analysis of the results was performed by using the BURST algorithm (http://www.mlst.net), which defines clonal complexes in which every isolate shares at least 5 identical alleles with at least 1 other isolate (Cox2, Cox5, Cox18, Cox20, Cox37, Cox56, and Cox57 were kept for the analysis) and characterizes ancestral genotypes. *C*. *burnetii* MST database was entered at the following website: http://ifr48.timone.univ-mrs.fr, and ST determination by sequence comparison is possible at this site.

## Results

### Choice of Spacers for Typing and Analysis by MST

Initially 14 isolates were chosen to test the genetic diversity of the spacers: Nine Mile, Priscilla, Q212, Heizberg, Brasov, Dog ut Ad, CB15, CB20, CB26, CB28, CB33, CB35, CB114, and CB115. We chose 68 spacers, but we retained only 51 spacers for which PCR amplification was obtained for all the isolates. We kept 10 spacers (Cox2, Cox5, Cox18, Cox20, Cox22, Cox37, Cox51, Cox56, Cox57, and Cox61) (Table 1) because they were representative of the results found when we analyzed the entire test set of 51 spacers. For each spacer, the number of variable sites in the sequences was determined, and the percentage of variability was calculated. They were, respectively, 1.1, 1.4, 1.9, 0.7, 2.3, 1.2, 1.4, 2.5, 1.7, and 2.1. We kept Cox18, Cox22, Cox51, Cox56, Cox57, and Cox61 because the percentage of variability in these spacers was high compared with the other spacers. We kept Cox2, Cox5, Cox20, and Cox37 because they allowed the characterization of CB35, CB15, CB26 and CB28, and Nine Mile respectively. To test the reliability of the spacers we kept, chi-square value was determined by using the value of 1% as the threshold value. The Fisher value was found to be statistically significant (9 × 10^–4^). We then added 159 other isolates. Sequences were obtained for all the isolates with spacers Cox2, Cox18, Cox20, Cox22, Cox37, Cox51, and Cox57. Mixed sequences were obtained with the isolate Poker Cat with spacers Cox5, Cox56, and Cox61. We cloned the PCR products and showed that several sequences were present after PCR amplification, including insertions or deletions. Allele distribution of the different gene spacers are described in Table 2. Each of the different sequences in a locus defined a distinct genotype, even if it differed from the others by only a single nucleotide. Thirty different sequence types (STs) were identified by using MST.

**Table 2:** Alleles of 10 spacers which allow the definition of the different Coxiella burnetii sequence types

COX	2	5	18	20	22	37	51	56	57	61
ST										
1	5	6	3	4	6	5	8	1	5	6
2	5	6	3	5	6	5	8	1	5	6
3	5	6	3	4	6	7	8	1	5	6
4	5	6	3	2	6	5	8	1	5	6
5	4	6	3	5	6	2	8	2	5	6
6	4	3	3	5	6	5	8	2	5	6
7	4	6	3	5	6	5	8	2	5	6
8	5	4	2	5	1	5	3	3	4	4
9	1	4	2	5	1	5	2	3	4	6
10	5	4	2	5	1	5	2	3	2	6
11	6	5	1	6	5	4	5	4	3	2
12	3	5	1	6	5	4	5	4	3	2
13	3	5	1	6	5	4	5	5	3	2
14	7	5	1	6	5	6	9	4	3	2
15	7	5	1	6	5	6	9	6	3	2
16	3	7	5	3	4	1	6	7	6	5
17	3	7	5	7	4	1	10	8	6	7
18	3	7	1	6	3	4	7	9	6	3
19	3	2	7	8	5	4	11	9	6	5
20	3	2	6	1	5	4	4	10	6	5
21	2	1	4	6	2	3	1	11	1	1
22	3	7	1	6	3	8	7	9	6	8
23	3	7	1	6	3	8	7	9	6	3
24	3	5	1	6	5	4	5	12	3	9
25	3	7	1	6	3	4	7	9	7	3
26	9	4	8	5	8	5	2	3	4	6
27	3	5	1	6	5	4	5	12	3	2
28	8	4	8	5	7	5	2	3	4	6
29	3	7	1	9	3	4	7	9	6	3
30	5	6	9	5	6	5	8	13	8	6

The nucleotide sequence accession numbers are noted in Table A3. Accession numbers for Poker Cat isolate clones are, respectively, AY619726, AY619728, and AY619729, and AY619721 for Cox5, Cox56, and Cox61.

### Computer Analysis of MST Data

The dendrogram in the Figure was constructed from a matrix of pairwise allelic differences between the compiled sequences of the 30 STs. We identified 3 monophyletic groups within the tree. The first group, representing 13 different STs, included isolates from France, Spain, Russia, Kyrgyzstan, Namibia, Kazakhstan, Ukraine, Uzbekistan, and the United States. It was divided in 2 subgroups. The first one included 36 isolates representing 8 different STs (ST1 to ST7 and ST30). Nineteen were represented by ST1. The second subgroup included 39 isolates which represented 5 different STs (ST8, ST9, ST10, ST26, and ST28). Twenty-eight were represented by ST8.

**Figure:**
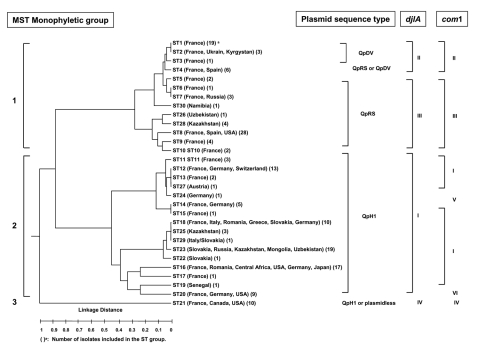
Dendrogram of the genetic relatedness among the 30 different sequence types defined by multispacer sequence type (MST) analysis. The dendrogram was constructed by unweighted pair-group method with arithmetic mean. Plasmid sequence type, *com1* group, and *djlA* group corresponding to each ST are indicated on the right of the figure. The 3 monophyletic groups defined by MST analysis are indicated on the left.

The second group included isolates from Europe (France, Germany, Switzerland, Romania, Italy, Greece, Austria, Slovakia), the United States, Russia, Africa (Central Africa and Senegal), and Asia (Kazakhstan, Uzbekistan, Mongolia, and Japan). It was divided into 4 subgroups. The first one included 26 isolates, which represented 7 different STs (ST11, ST12, ST13, ST14, ST15, ST24, and ST27). The second subgroup included 34 isolates that were included in ST18, ST22, ST23, ST25, and ST29 groups. The third subgroup included 18 isolates (ST16 and ST17), and the fourth subgroup included 10 isolates (ST19 and ST20).

The third group consisted of only 1 ST, ST21, and included the 7 Canadian isolates, 2 isolates from France (CB4 and CB7), and 1 isolate from the United States (Scurry). The clusters determined by the BURST algorithm were consistent with those determined by the phylogenetic analysis. Five groups were defined. The first one included ST1 to ST7; the putative ancestral genotype in this group was ST1. ST8 (putative ancestral genotype), ST9, ST10, ST26, and ST28 were included in the second group; ST11, ST12 (putative ancestral genotype), ST13, ST14, ST15, and ST24 in the third group, ST16 and ST17 in the fourth group; and ST18 (putative ancestral genotype), ST22, ST23, ST25, and ST29 in the fifth group. ST19, ST20, ST21, and ST30 were considered as singletons.

### Sequence Type Determination and Correlation with Pathology

In the monophyletic group 1, the sequence of plasmid QpRS was found for isolates included in ST4, ST5, ST6, ST7, ST8, ST9, ST10, ST26, ST28, and ST30. The QpDV plasmid sequence was amplified for isolates included in ST1, ST2, ST3, and ST4. In the monophyletic group 2, the QpH1 plasmid sequence was found in all the isolates. In the monophyletic group 3, the QpH1 plasmid sequence or none of the searched plasmid sequences was detected. Sequence comparison of *djlA* generated 4 different groups. Group I included all STs included in the monophyletic group 2 defined by MST analysis. Group II included ST1, ST2, ST3, and ST4. Group III included ST5, ST6, ST7, ST30, ST26, ST28, ST8, ST9, and ST10. Group IV corresponded to ST21. *Com1* sequence comparison generated 6 different groups. Group I included all the STs included in the monophyletic group 2 defined by MST analysis except ST14 (group V) and ST20 (groupVI). Group II included ST1, ST2, ST3, and ST4. Group III included ST5, ST6, ST7, ST30, ST26, ST28, ST8, ST9, and ST10. Group IV corresponded to ST21. When *com1* typing was used, only 1 strain was not in accordance with MST typing results. This strain, CB95, was included in ST8 but exhibited a group II *com1* sequence.

QpDV plasmid presence in human isolates was correlated with the acute form of the disease (p = 2 × 10^–7^), and QpRS plasmid presence was correlated with the chronic form of the disease (p = 2 × 10^–4^). The acute form of the disease was correlated with ST1 (p = 10^–3^), ST4 (p = 7 × 10^–4^) ST16 (p = 3 × 10^–3^), ST18 (p = 10^–2^), and the chronic form of the disease was correlated with ST8 (p = 2 × 10^–3^).

### Modifications in ORFs Surrounding Studied Spacers

As primers were chosen in ORFs surrounding the studied spacers, mutations, deletions, or insertions were noted in the protein sequences. Mutations were noted in the hypothetical protein (gi29653385) for ST11; in the hypothetical protein (gi29653385) for ST9 and ST26; in entericin (gi29653446) for ST20, in ribonuclease H (gi29653667) in ST1, 2, 3, 4, 5, 6, 7, 8, 9, 10, 21, 26, 28, and 30; in amino acid permease family protein (gi29653908) in ST28; in hypothetical protein (gi29654047) in ST1, 2, 4, 5, 6, 7, 8, 9, 10, 26, 28, and 30. In CB118 (ST3), a stop codon appeared which shortened the length of the ORF. Mutations were noted in uridine kinase (gi29654198) in ST18, ST22, ST23, ST25, and ST29; in ompA-like transmembrane domain protein (gi29654257), in ST20; in rhodanese-like domain protein (gi29654263) in ST20 (the protein was longer by 2 amino acids); in dioxygenase (gi29654325) in ST21 and ST22; in hypothetical protein (gi29732244), in ST17.

Insertions or deletions were noted in hypothetical protein (gi29653386) in ST5, 6, and 7; in hypothetical protein (gi29653755) in ST1 and ST3 (insertion of a base G in the DNA sequence made the protein sequence longer of 22 amino acids); in the amino acid permease family protein (gi29653772) in ST8, 9, and 10 (deletion of a base A in the DNA sequence made the protein sequence longer of 24 amino acids); in ompA-like transmembrane domain protein (gi29654257) in ST11, 12, 13, 14, 15, 16, 17, 18, 19, 20, 22, 23, 24, 25, 27, and 29.

## Discussion

Q fever in humans and animals, caused by *C*. *burnetii*, is found worldwide. In humans, it causes a variety of diseases such as acute flulike illness, pneumonia, hepatitis, and chronic endocarditis. In animals, *C*. *burnetii* is found in the reproductive system, both uterus and mammary glands and may cause abortion or infertility.

Molecular methods are now almost universally used to characterize strains and to determine the relatedness between isolates causing diseases in different contexts. The most discriminative approach used for *C*. *burnetii* isolates until this study was PFGE. Twenty different restriction patterns were distinguished after *Not*I restriction of total *C*. *burnetii* DNA and PFGE (11). Comparison of PFGE profiles is sometimes difficult because good separation of the different fragments is required. For example, the isolate Heizberg was classified in group 1 by Thiele et al. (10) and in group 2 by Jäger et al. (11). This fact highlights the difficulty of comparing results obtained by this technique. Moreover, in some species, rapid genomic rearrangements occur because of repeats or insertion sequences, so even if isolates descended from a common ancestor that arose several decades ago, they may not readily be seen to be minor variants of the same clone. In these cases, PFGE does not contribute to tracing of isolates. The great advantage of MST over PFGE as a typing method is the lack of ambiguity and the portability of sequence data, which allow results from different laboratories to be compared without exchanging strains. This work is the first to include so many isolates in a rigorous examination of molecular epidemiology. The study of this bank of sequences will contribute to understanding the propagation mode of the bacteria as variations accumulate relatively slowly, thus making it an ideal tool for global epidemiology. For example, in ST16 we characterized isolates that were obtained from 1935 (Nine Mile) to 1991 (CB25).

Most of the French isolates were included in monophyletic group 1. Nineteen were included in ST1, and 24 were included in ST8. Thus, an isolate has a geographic distribution even if genetic modifications appear (insertions, deletions or mutations) over time, giving rise to a new ST that is related to the ancestor isolate. This fact was highlighted when the analysis of the STs was performed by using the BURST algorithm. ST1 and ST8 were described as the ancestral genotypes and for example, ST9 and ST10 corresponded to SLVs of ST8 (isolates that differ at only 1 of the 7 loci) and ST26 and ST28 corresponded to DLVs of ST8 (double locus variants). But some types were not delineated on the basis of geographic origin because they were isolated from different parts of the world. This distribution in distant countries is likely related to movements of infected patients, animals, or ticks. This is particularly true for ST16 isolates that were encountered on 4 different continents, America, Europe, Asia, and Africa. The homology of the Canadian isolates from Nova Scotia should be noted. Q fever is just as endemic in Nova Scotia as in France. This may indicate rapid and recent spreading of a single strain. The association between ST21 and Canada is significant as tested with the chi-square test with a Fisher value <10^–8^. Notably, patient CB115, who had Q fever endocarditis, was living in Edmonton, Alberta (≈3,000 miles from Nova Scotia) when this illness was diagnosed. He grew up in Nova Scotia, and the molecular epidemiologic findings show that he acquired his disease there. Q fever is uncommon in Alberta. Most of the STs are found in Europe. A sample bias could exist as most of the isolates tested were from this continent, but the results obtained may also indicate that *C. burnetii* originated from the Old World and spread later in the New World, excluding New Zealand.

Concordant results were found when MST was compared with *com1* and *djlA* sequences comparison (Figure) However MST was more discriminant. Plasmid profile investigation of *C*. *burnetii* detected 4 different plasmids QpH1, QpRS, QpDV, and QpDG and 1 group of plasmidless isolates. QpH1 was first found in the Nine Mile tick isolate (28). QpRS was first found in the goat isolate Priscilla (29). QpDG was described from isolates obtained from feral rodents near Dugway, Utah (8). QpDV was found in French and Russian isolates (5,6). Another not-well-characterized plasmid type was described in China (30). The existence of a plasmidless *C*. *burnetii* isolate, Scurry Q217 was described (31), but a chromosomally integrated plasmid-homologous DNA fragment was found in this isolate by hybridization (32,33). Plasmid type sequence detection was also correlated with MST. Group 2 included isolates that PCR amplification found to be positive with primers specific for QpH1. Group 3 included 3 isolates, 2 from France (CB4 and CB7) and 1 from Nova Scotia (Poker Cat), in which plasmid sequence type of QpH1 was detected. No such sequence was detected in the other isolates of Nova Scotia origin included in group 3. Group 1 included isolates that were positive by PCR amplification with primers specific for QpRS (47/77). QpDV plasmid was described in isolates from France, Spain, Ukraine, and Kyrgyzstan. In fact, regions shared by QpH1, QpRS, and QpDV were termed "core plasmid sequences" and encompassed 25 kb. QpH1, QpRS, and QpDV are, respectively, 37 kb, 39 kb, and 33 kb in size. Integrated sequences in American isolate represent 18 kb. Differences in plasmid size and sequence can be explained by notable sequence rearrangements, such as deletions, insertions, or duplications, because several repeat sequences have been identified through which such rearrangements might have occurred. For CB13, we were able to characterize sequences for plasmids QpH1 and QpDV, which can be caused by several situations: this isolate may have 1) 2 different plasmids, 2) a QpH1 plasmid and sequences of QpDV integrated in the chromosome, or 3) a new plasmid that arose from combination of QpH1 and QpDV. All these hypotheses are in agreement with the presence of QpH1 plasmid in the ancestor of *C. burnetii* isolates. This plasmid was lost by some of them (monophyletic group 3) but genetic information of crucial importance for the organism was integrated in the chromosome. For other isolates, QpH1 plasmid evolved to QpRS plasmid, in some isolates QpRS plasmid evolved to QpDV plasmid.

This study showed a correlation between QpDV and acute infections, between QpRS and chronic infections, and an association between some genotypes and disease type. A bias in sampling exists since acute disease is 20 times more frequent than chronic disease, but in this study, most of the human isolates were from chronic disease patients, and the isolates from acute infections were mainly obtained from France. These facts reflect the difficulty in isolating the bacteria. A genomic typing method such as MST could be applied directly to samples to obtain a more precise idea of how *C*. *burnetii* is spreading in the environment and the pathogenetic implications in acute and chronic forms of Q fever.

Comparison of DNA sequences is the best approach to investigate bacterial evolution. MLST in association with BURST analysis has been used to type isolates of many species. But this method is useful only if housekeeping gene diversity exists in the studied species. For example, in the species *Yersinia pestis* no diversity was found in the housekeeping genes studied (34). With the MST approach, differentiation of the 3 biovars Antiqua, Medievalis, and Orientalis was possible (25), which shows that the discriminatory power of MST is higher than that of MLST and is comparable to that of tandem repeats analysis (35). Low variability was found in *C*. *burnetii* housekeeping genes such as 16S rRNA (36) and *rpoB* (37). MST is the first method that allows a rapid and reliable typing of *C*. *burnetii* isolates during investigations of outbreaks by sequencing the PCR product obtained from the 10 spacers described. We did not test isolates from Australia and only 8 from the United States. Two isolates from Africa (Namibia and CB119) were considered as singletons in the BURST analysis denoting lack of closely related isolates. In the future, isolates that were not available in our laboratory during this study must be tested so the missing links in our phylogenetic analysis can be determined. The constitution of a database in a website will allow isolates from all the countries in the world to be compared and increase understanding of the propagation of the isolates of *C*. *burnetii*.

## Appendix

**Table A1:** Isolates of Coxiella burnetii studied

Isolate	Origin	Disease, symptoms, clinical status	Geographicsource	ST (sequence type)	Plasmid sequence type
CB108	Human blood	Acute	Marseille, France, 2001	1	QpDV
CB1	Human heart valve	Chronic	Istres, France, 1989	1	QpDV
CB3	Human heart valve	Chronic	Marseille, France	1	QpDV
CB36	Human placenta	Abortion	Martigues, France, 1992	1	QpDV
CB38	Human blood	Acute	Marseille, France, 1992	1	QpDV
CB41	Human heart valve	Chronic	Marseille, France, 1993	1	QpDV
CB60	Human blood	Acute	Marseille, France, 1996	1	QpDV
CB63	Human heart valve	Chronic	Marseille, France, 1994	1	QpDV
CB75	Human blood	Chronic	Marseille, France, 1998	1	QpDV
CB82	Human blood	Acute	Marseille, France, 1999	1	QpDV
CB86	Human blood	Chronic	Marseille, France, 1999	1	QpDV
CB87	Human placenta	Abortion	Martigues, France, 1999	1	QpDV
CB89	Human placenta	Abortion	Martigues, France, 2000	1	QpDV
CB94	Human blood	Acute	Aix en Provence, France, 2000	1	QpDV
CB97	Human blood	Acute	Marseille, France, 2000	1	QpDV
CB110	Human blood	Chronic	Marseille, France, 2002	1	QpDV
CB28	Human blood	Acute	Salon de Provence, France, 1992	1	QpDV
CB26	Human blood	Acute	Marseille, France, 1992	1	QpDV
CB64	Human blood	Acute	Martigues, France, 1996	1	QpDV
CB39	Human blood	Acute	Marseille, France, 1992	2	QpDV
RT-1140	Human blood	Pneumonia	Krimea, Ukrain 1954	2	QpDV
RT-Schperling	Human blood	Fever	Kyrgyzstan, 1955	2	QpDV
CB118	Human heart valve	Chronic	Marseille, France, 2004	3	QpDV
CB62	Human blood	Acute	Martigues, France, 1996	4	QpDV
CB20	Human blood	Acute	Salon de Provence, France, 1991	4	QpRS
CB51	Human placenta	Abortion	Madrid, Spain, 1996	4	QpDV
CB12	Human blood	Acute	Aix en Provence, France	4	QpDV
CB57	Human blood	Acute	Martigues, France, 1996	4	QpDV
CB54	Human blood	Acute	Aix en Provence, France, 1996	4	QpDV
CB111	Human heart valve	Chronic	Marseille, France, 2003	5	QpRS
CB35	Human heart valve	Chronic	Paris, France, 1992	5	QpRS
CB45	Human heart valve	Chronic	Paris, France, 1993	6	QpRS
CB43	Human heart valve	Chronic	Paris, France, 1993	7	QpRS
Leningrad-2	Human blood	-	Leningrad, Russia, 1955	7	QpRS
Leningrad-4	Human blood	-	Leningrad, Russia, 1957	7	QpRS
CB10	Human heart valve	Chronic aneurysm	Grenoble, France	8	QpRS
CB9	Human blood	Chronic	Lyon, France	8	QpRS
CB31	Human heart valve	Chronic	Marseille, France, 1992	8	QpRS
CB34	Human blood	Chronic	Marseille, France, 1992	8	QpRS
CB44	Human heart valve	Chronic	Créteil, France, 1993	8	QpRS
CB53	Aneurysm	Chronic	Marseille, France, 1995	8	QpRS
CB61	Valvular prosthesis	Chronic	Marseille, France, 1996	8	QpRS
CB70	Human heart valve	Chronic	Grenoble, France, 1997	8	QpRS
CB71	Valvular prosthesis	Chronic	Saint-Laurent du Var, France, 1997	8	QpRS
CB73	Valvular prosthesis	Chronic	Marseille, France, 1998	8	QpRS
CB81	Human heart valve	Chronic	Madrid, Spain, 1999	8	QpRS
CB91	Valvular prosthesis	Chronic	Marseille, France, 2000	8	QpRS
CB93	Human placenta	Abortion	Dreux, France, 2000	8	QpRS
CB95	Human blood	Chronic	Marseille, France, 2000	8	QpRS
CB116	Aneurysm	Chronic	Marseille, France, 2003	8	QpRS
CB107	Human heart valve	Chronic	Tours, France, 2001	8	QpRS
CB96	Human heart valve	Chronic	Marseille, France, 2000	8	QpRS
CB114	Human heart valve	Chronic	Marseille, France, 2003	8	QpRS
CB15	Human heart valve	Chronic	Lyon, France, 1991	8	QpRS
CB47	Human heart valve	Chronic	Barcelone, Spain, 1994	8	QpRS
CB99	Human heart valve	Chronic	Marseille, France, 2000	8	QpRS
CB106	Human heart valve	Chronic	Toulouse, France, 2001	8	QpRS
CB79	Human heart valve	Chronic	Paris, France, 1999	8	QpRS
CB98	Human heart valve	Chronic	Marseille, France, 2000	8	QpRS
CB83	Goat placenta	Abortion	Newfoundland, USA, 1999	8	QpRS
CB8	Human heart valve	Chronic	Marseille, France, 1990	8	QpRS
Priscilla	Aborted goat	Abortion	Montana, USA, 1980	8	QpRS
CB117	Human heart valve	Chronic	Marseille, France, 2004	8	QpRS
CB32	Human heart valve	Chronic	Lyon, France, 1992	9	QpRS
CB92	Human heart valve	Chronic	Marseille, France, 2000	9	QpRS
CB68	Pigeon excrement	–	Marseille, France, 1996	9	QpRS
CB49	Human heart valve	Chronic	Marseille, France, 1994	9	QpRS
CB65	Human heart valve	Chronic	Marseille, France, 1996	10	QpRS
CB103	Ewe placenta	Abortion	Marseille, France, 2001	10	QpRS
CB13	Human blood	Chronic	Paris, France	11	QpH1/QpDV
CB40	Human heart valve	Chronic	Paris, France, 1993	11	QpH1
CB46	Valvular prosthesis	Chronic	Paris, France, 1993	11	QpH1
CB5	Human blood	Chronic	Paris, France, 1990	12	QpH1
CB6	Human blood	Chronic	Paris, France	12	QpH1
CB42	Valvular prosthesis	Chronic	Toulouse, France, 1993	12	QpH1
CB52	Valvular prosthesis	Chronic	Paris, France, 1995	12	QpH1
CB56	Human heart valve	Chronic	Paris, France, 1996	12	QpH1
CB58	Spleen abscess	–	Lyon, France, 1996	12	QpH1
CB76	Human heart valve	Chronic	Paris, France, 1998	12	QpH1
CB105	Human heart valve	Chronic	Montpellier, France, 2001	12	QpH1
CB112	Human heart valve	Chronic	Zurich, Switzerland, 2003	12	QpH1
CB109	Human heart valve	Chronic	Berlin, Germany, 2002	12	QpH1
CB113	Goat placenta	Abortion	Albi, France, 2003	12	QpH1
CB33	Human heart valve	Chronic	Clermont-Ferrand, France, 1992	12	QpH1
CB55	Vegetation	Chronic	Paris, France, 1996	12	QpH1
CB2	Human blood	Immunodepression	Toulouse, France,	13	QpH1
CB69	Vegetation	Chronic	Toulouse, France, 1996	13	QpH1
CB85	Human blood	Chronic	Tours, France, 1999	14	QpH1
CB74	Valvular prosthesis	Chronic	Toulouse, France, 1998	14	QpH1
CB59	Aneurysm	Chronic aneurysm	Saint-Etienne, France, 1996	14	QpH1
CB80	Node	Chronic	Niort, France, 1999	14	QpH1
Z3055	Ewe placenta	Abortion	Germany	14	QpH1
CB102	Valvular prosthesis	Chronic	Poitiers, France, 2001	15	QpH1
CB11	Human blood	Acute	Marseille, France	16	QpH1
CB23	Human blood	Chronic	Clermont-Ferrand, France, 1988	16	QpH1
Brasov	Human	Acute	Romania	16	QpH1
Bangui	Human blood	Acute	Central Africa	16	QpH1
California	Cow milk	Persistent	California, USA, 1947	16	QpH1
CB25	Human blood	Acute	Paris, France, 1991	16	QpH1
Dyer	Human blood	Acute	USA, 1938	16	QpH1
Ohio	Cow milk	Persistent	Ohio, USA, 1956	16	QpH1
Nine Mile	Tick	–	Montana, USA, 1935	16	QpH1
CS-KL 9	Ixodes ricinus	–	Slovakia, 1989	16	QpH1
Z-2775/90	Cow placenta	Abortion	Germany, 1990	16	QpH1
J-1	Cow milk	–	Japan	16	QpH1
J-3	Cow milk	–	Japan	16	QpH1
J-27	Cow milk	–	Japan	16	QpH1
J-60	Cow milk	–	Japan	16	QpH1
J-82	Cow milk	–	Japan	16	QpH1
Hardthof	Cow milk	–	Germany, 1990	16	QpH1
CB77	Human heart valve	Chronic	Paris, France, 1998	17	QpH1
CB100	Human blood	Chronic	Strasbourg, France	18	QpH1
Henzerling	Human blood	Acute	Italy/Slovakia, 1945	18	QpH1
Balaceanu	Human	Acute	Romania	18	QpH1
Geier	Human	Acute	Romania	18	QpH1
Heizberg	Human	Acute	Greece	18	QpH1
Cs-Florian	Human blood	–	Slovakia, 1956	18	QpH1
Z-3464/92	Goat placenta	Abortion	Germany, 1992	18	QpH1
Z-4488/93	Ewe placenta	Abortion	Germany, 1993	18	QpH1
Z-349-36/94	Ewe placenta	–	Germany, 1994	18	QpH1
München	Sheep	–	München, Germany, 1969	18	QpH1
CB119	Human heart valve	Chronic	Senegal, 2004	19	QpH1
CB48	Human placenta	Abortion	Grenoble, France, 1994	20	QpH1
CB50	Valvular prosthesis	Chronic	Paris, France, 1994	20	QpH1
CB66	Aneurysm	Chronic aneurysm	Marseille, France, 1996	20	QpH1
CB72	Valvular prosthesis	Chronic	Paris, France, 1996	20	QpH1
CB78	Valvular prosthesis	Chronic	Marseille, France, 1998	20	QpH1
CB88	Human heart valve	Chronic	Lyon, France, 1999	20	QpH1
CB90	Human heart valve	Chronic	Lyon, France,2000	20	QpH1
Z-3567/92	Cow placenta	Abortion	Germany, 1992	20	QpH1
Dugway 5J108-111	Rodent	–	Utah, USA, 1958	20	QpH1
CB4	Human blood	Chronic	Montpellier, France,1988	21	QpH1
CB7	Human heart valve	Chronic Aneurysm	Marseille, France	21	QpH1
Q229	Human heart valve	Chronic	Nova Scotia, Canada, 1982	21	QpH1
Dog CB	Dog uterus	–	Nova Scotia, Canada, 1995	21	
Poker Cat	Cat	–	Nova Scotia, Canada, 1986	21	
CBNSC1	Cat	–	Nova Scotia, Canada, 1986	21	QpH1
Dog ut Ad	Dog uterus	–	Nova Scotia, Canada, 1989	21	
CB115	Human heart valve	Chronic	Nova Scotia, Canada, 2003	21	
Q212	Human heart valve	Chronic	Nova Scotia, Canada, 1981	21	
Scurry Q217	Human liver biopsy	Hepatitis	Rocky Mountain, USA	21	
48	*Haemaphysalis punctata*	–	Slovakia, 1970	22	QpH1
Irkutsk	Tick	–	Irkutsk, Russia1969	23	QpH1
Uzbekistan	Cow placenta	–	Uzbekistan, 1971	23	QpH1
1894	Liver and spleen of wild bird	–	Czechoslovakia, 1954	23	QpH1
Kazakhstan-6	*Dermacentor marginatus*	–	Kazakhstan, 1969	23	QpH1
K-261-Louga	Cow milk	–	Leningrad, Russia, 1959	23	QpH1
Kazakhstan -5	*Dermacentor hirundinis*	–	Kazakhstan, 1969	23	QpH1
Russet mouse	Russet mouse viscera	–	Pskov, Russia	23	QpH1
II/IA	*Dermacentor marginatus*	–	Slovakia, 1972	23	QpH1
IXO	*Ixodes ricinus*	–	Czech Republic, 1957	23	QpH1
Mongolia-2	*Dermacentor nuttalli*	–	Mongolia, 1985	23	QpH1
CS-27	*Dermacentor marginatus*	–	Slovakia, 1972	23	QpH1
Oufa-1	Human blood	–	Oufa, Russia	23	QpH1
Oufa-2	Ewe placenta	Abortion	Oufa, Russia	23	QpH1
Louga-3	*Ixodes ricinus*	–	Leningrad, Russia, 1962	23	QpH1
Louga-2	*Ixodes ricinus*	–	Leningrag, Russia, 1959	23	QpH1
Louga-1	*Cimex lecturalius*	–	Leningrad, Russia, 1959	23	QpH1
Louga rodent	*Apodemus flavicollis* viscera	–	Leningrad, Russia, 1958	23	QpH1
Mongolia-1	*Dermacentor silvarum*	–	Mongolia, 1984	23	QpH1
Vologda-2	Human blood	–	Vologda, Russia, 1987	23	QpH1
8931 F10	Cow placenta	Abortion	Germany	24	QpH1
Grita	-	–	Germany, 1940-1945	25	QpH1
M-44	Vaccine	–	Russia	25	QpH1
Kazakhstan-4	Fly	–	Kazakhstan, 1965	25	QpH1
Termez	Human blood	–	Uzbekistan, 1952	26	QpRS
Z2534	Goat placenta	Abortion	Austria	27	QpH1
Kazakhstan-1	Ewe placenta	Abortion	Kazakhstan	28	QpRS
Kazakhstan-2	Cow milk	–	Kazakhstan, 1962	28	QpRS
Kazakhstan-3	*Hyalomma* tick	–	Kazakhstan, 1962	28	QpRS
Tcheredov	Human blood	–	Kazakhstan, 1965	28	QpRS
Henzerling-r *	Human blood	–	Italy, 1945 / Slovakia,	29	QpH1
Namibia	Goat	–	Namibia, 1991	30	QpRS

*Strain resistant to chlortetracycline obtained by passages in embryonated hen's eggs in presence of increasing doses of chlortetracycline.

**Table A2:** Primer used in djlA, com1 and QpH1 and QpRS plasmid targeted sequences for PCR amplification and sequencing

Primer	Nucleotide sequence 5´ → 3´	Position in the gene	
djlA-1*†	CGGTGATGAACTGGATTGG	–5 – 15	
djlA-2†	ATTGACCTGACGCGCTTGACG	266 –247	
djlA-3†	GGCAACGCAAGACCCCCGTG	579 – 598	
djlA-4*†	AACCATGCTTCGCACCTTAC	810 –791	
com-1*†	CGTGAAGAACCGTTTGACTG	3 – 22	
com-2†	TGAGGATTGCCTGCCACTGG	284 – 265	
com-3†	GCGCTGCTCAGTGTCGACGG	490 – 509	
com-4*†	CTTTTCTACCCGGTCGATTTC	759 – 739	
Primer	Nucleotide sequence 5´ → 3´	Position in the plasmid	ORF
QpH11	TGACAAATAGAATTTCTTCATTTTGATG	QpH1 (gb:AE016829) 15332 –15359	Spacer between two hypothetical proteins
QpH12	GCTTATTTTCTTCCTCGAATCTATGAAT	QpH1 (gb:AE016829) 168348 – 16375	Spacer between two hypothetical proteins
QpRSO1	CTCGTACCCAAAGACTATGAATATATCC	QpRS gb:Y15898) 14761 –14734	Hypothetical protein
QpRS02	CACATTGGGTATCGTACTGTCCCT	QpRS gb:Y15898) 14398 – 14321	Hypothetical protein
QpDV1f	ATGAGAGAAGAGCAGCCGCT	QpRS (gb:Y15898) 9889 – 9908	Hypothetical protein
QpDV1r	TCAATGATCCGATGTGCGTTT	QpH1 (gb:Y15898) 10634 –10614	Hypothetical protein

*Oligonucleotide primer used for PCR amplification. †Oligonucleotide primer used for sequencing.

**Table A3:** Accession numbers of nucleotide sequences deposited in GenBank

	Cox 2	Cox 5	Cox 18	Cox 20	Cox 22	Cox 37	Cox 51	Cox 56	Cox 57	Cox 61
ST1 (CB26)	AY492067	AY495357	AY502819	AY502857	AY502899	AY502623	AY502735	AY502777	AY502674	AY512785
ST2 (CB39)	AY574327	AY574328	AY574329	AY574330	AY574331	AY574332	AY574333	AY574334	AY574335	AY574336
ST3 (CB118)	AY574337	AY574338	AY574339	AY574340	AY574341	AY574342	AY574343	AY574344	AY574345	AY574346
ST4 (CB20)	AY492065	AY495355	AY502817	AY502855	AY502897	AY502621	AY502733	AY502775	AY502673	AY512784
ST5 (CB35)	AY494735	AY495360	AY502822	AY502860	AY502902	AY502626	AY502738	AY502780	AY502677	AY512788
ST6 (CB45)	AY494734	AY502720	AY502841	AY502881	AY502921	AY502645	AY502761	AY502801	AY502709	AY512819
ST7 (CB43)	AY574307	AY574308	AY574309	AY574310	AY574311	AY574312	AY574313	AY574314	AY574315	AY574316
ST8 (Priscilla)	AY596174	AY502715	AY502837	AY502875	AY502883	AY502642	AY502752	AY502797	AY502681	AY596175
ST9 (CB68)	AY494733	AY502721	AY502842	AY502882	AY502922	AY502646	AY502762	AY502802	AY502710	AY512820
ST10 (CB103)	AY492058	AY495348	AY502810	AY502850	AY502890	AY502613	AY502728	AY502770	AY502688	AY512805
ST11 (CB13)	AY575668	AY575669	AY575670	AY575671	AY575672	AY575673	AY575674	AY575675	AY575676	AY575677
ST12 (CB33)	AY492069	AY495359	AY502821	AY502859	AY502901	AY502625	AY502737	AY502779	AY502676	AY512787
ST13 (CB2)	AY575653	AY575654	AY575655	AY575656	AY575657	AY575658	AY575659	AY575660	AY575661	AY575662
ST14 (CB85)	AY575643	AY575644	AY575645	AY575646	AY575647	AY575648	AY575649	AY575650	AY575651	AY575652
ST15 (CB102)	AY492057	AY495347	AY502809	AY502849	AY502889	AY502612	AY502755	AY502769	AY502687	AY512794
ST16 (Ohio)	AY494725	AY502712	AY502834	AY502872	AY502916	AY502640	AY502748	AY502794	AY502707	AY512816
ST17 (CB77)	AY574325	AY574326	AY574317	AY574318	AY574319	AY574320	AY574321	AY574322	AY574323	AY574324
ST18 (Henzerling)	AY494723	AY495369	AY502832	AY502870	AY502914	AY502638	AY502746	AY502792	AY502701	AY512814
ST19 (CB119)	AY575633	AY575634	AY575635	AY575636	AY575637	AY575638	AY575639	AY575640	AY575641	AY575642
ST20 (CB88)	AY492072	AY502719	AY502825	AY502863	AY502905	AY502629	AY502759	AY502783	AY502696	AY512798
ST21 (Q212)	AY494729	AY502716	AY502838	AY502876	AY502918	AY502643	AY502753	AY502798	AY502682	AY512793
ST22 (48)	AY864229	AY864230	AY864231	AY864232	AY864233	AY864234	AY864235	AY864236	AY864237	AY864238
ST23 (Irkutsk)	AY864239	AY864240	AY864241	AY864242	AY864243	AY864244	AY864245	AY864246	AY864247	AY864248
ST24 (8931 F10)	AY864199	AY864200	AY864201	AY864202	AY864203	AY864204	AY864205	AY864206	AY864207	AY864208
ST25 (Grita)	AY864209	AY864210	AY864211	AY864212	AY864213	AY864214	AY864215	AY864216	AY864217	AY864218
ST26 (Termez)	AY864219	AY864220	AY864221	AY864222	AY864223	AY864224	AY864225	AY864226	AY864227	AY864228
ST27 (Z2534)	AY864249	AY864250	AY864251	AY864252	AY864253	AY864254	AY864255	AY864256	AY864257	AY864258
ST28 (Kazakhstan-2)	AY864259	AY864260	AY864261	AY864262	AY864263	AY864264	AY864265	AY864266	AY864267	AY864268
ST29 (Henzerling-r)	AY864269	AY864270	AY864271	AY864272	AY864273	AY864274	AY864275	AY864276	AY864277	AY864278
ST30 (Namibia)	AY864279	AY864280	AY864281	AY864282	AY864283	AY864284	AY864285	AY864286	AY864287	AY864288
